# Quercetin Attenuates Non-Alcoholic Fatty Liver Disease in Association with the Inhibition of Hepatic IL-1β/iNOS and IL-1β/CD45 Axes of Inflammation and Fibrosis Accompanied by Reduced Endogenous Metabolites and Apoptosis

**DOI:** 10.3390/metabo16040284

**Published:** 2026-04-21

**Authors:** Saif A. Alqahtani, Hanan H. Alshehri, Hend Ashour, Hend Abdallah, Laila Rashed, Rehab M. Badi, Muataz E. D. Mohammed, Bahjat Al-Ani, Norah M. Alzamil, Alia Albawardi, Basma E. Aboulhoda

**Affiliations:** 1 Internal Medicine Department, College of Medicine, King Khalid University, Abha 61421, Saudi Arabia; saif@kku.edu.sa (S.A.A.); hhsalim@kku.edu.sa (H.H.A.); 2 Department of Physiology, College of Medicine, King Khalid University, Abha 61421, Saudi Arabia; hiahmad@kku.edu.sa (H.A.); rbadi@kku.edu.sa (R.M.B.); dmohamed@kku.edu.sa (M.E.D.M.); balani@kku.edu.sa (B.A.-A.); 3 Department of Anatomy, College of Medicine, King Khalid University, Abha 61421, Saudi Arabia; hbadawi@kku.edu.sa; 4 Department of Anatomy and Embryology, Faculty of Medicine, Cairo University, Cairo 12613, Egypt; hend.badawy@kasralainy.edu.eg; 5 Department of Medical Biochemistry and Molecular Biology, Faculty of Medicine, Cairo University, Cairo 12613, Egypt; lailarashed@kasralainy.edu.eg; 6 Department of Family and Community Medicine, College of Medicine, Princess Nourah Bint Abdulrahman University, P.O. Box 84428, Riyadh 11671, Saudi Arabia; nmalzamil@pnu.edu.sa; 7 Department of Pathology, College of Medicine and Health Sciences, United Arab Emirates University, Al Ain P.O. Box 64141, United Arab Emirates

**Keywords:** NAFLD, MASLD, IL-1β, iNOS, CD45, inflammation, liver fibrosis, p53, quercetin

## Abstract

Background: Liver inflammation and fibrosis are directly associated with non-alcoholic fatty liver disease (NAFLD). Dysregulation of the potent pro-inflammatory cytokine interleukin-1 beta (IL-1β), inducible nitric oxide synthase (iNOS), and tissue leukocyte infiltration (CD45 +ve) are connected with multiorgan injury and fibrosis. We investigated whether the induction of NAFLD can cause dysregulation in the hepatic IL-1β/iNOS and IL-1β/CD45 axes of inflammation and fibrosis, as well as in endogenous metabolites (lipids, glucose, and insulin) and apoptosis, in the presence and absence of the flavonoid quercetin. Methods: The model group of rats was fed with a high-fat and high-carbohydrate diet (HFCD) for 4 weeks. The protective group of rats was given both quercetin (50 mg/kg) and HFCD for 4 weeks. All rats were sacrificed on day 29. Results: NAFLD was induced in rats as demonstrated by dyslipidemia, hyperglycemia, insulin resistance, liver inflammation, and elevation of liver injury enzymes. NAFLD was also associated with the upregulation of hepatic IL-1β, iNOS, CD45, and apoptosis (p53). Biomarkers of fibrosis (TIMP-1 and α-SMA) were also elevated, and fibrosis was confirmed in the model group by increased collagen deposition and elevated stages of fibrosis score (Stage 1 to 2 of Brunt’s NASH classification). All these parameters were significantly (*p* < 0.01) modulated by quercetin treatment. Additionally, a significant (*p* < 0.001) correlation between IL-1β and hepatic injury parameters was observed. Conclusions: These findings suggest a potential association between NAFLD and the IL-1β/iNOS and IL-1β/CD45 axes of liver injury and fibrosis, as well as dyslipidemia, glycemia, and apoptosis, with quercetin exhibiting beneficial hepatic pleiotropic effects.

## 1. Introduction

Accumulation of fat (triglycerides) inside the major cells of the liver (hepatocytes) and insulin resistance lead to NAFLD (also known as metabolic dysfunction-associated steatotic liver disease [MASLD]), which represents the metabolic syndrome of the liver [1]. The continuous fat buildup in the liver can progress from a simple steatosis (fatty liver) to a more complicated condition called non-alcoholic steatohepatitis (NASH) associated with inflammation, liver scarring (fibrosis and cirrhosis), and liver failure, as well as liver cancer in advanced cases [2]. Type 2 diabetes mellitus is a serious complication of metabolic syndrome and is associated with NAFLD and obesity; one study reported that 68% of diabetic patients diagnosed with NAFLD, which significantly increased the incidence of liver fibrosis and mortality rates related to NASH, associated with the rising prevalence of obesity and type 2 diabetes [3,4]. Indeed, the World Obesity Federation warns that the number of people living with obesity is set to reach one billion by 2030, and excess adipose tissue produces metabolic inflammation that leads to insulin resistance and other metabolic disorders like NAFLD [5]. Additionally, there is a positive feedback loop between NAFLD and gestational diabetes mellitus that promotes systemic inflammation [6,7]. Therefore, investigating hepatic inflammatory cell signaling pathways in NAFLD and NASH is valuable because inflammation is a key driver of disease progression [8], and targeting these pathways can provide therapeutic potential.

Dysregulation of the intracellular multiprotein complexes (inflammasomes) triggers inflammation by maturing and releasing pro-inflammatory cytokines like IL−1β [9], causing tissue damage in metabolic disorders such as diabetes mellitus [10], acute gout (hyperuricemia) flares in adult patients [11], obesity [12], and NAFLD progression [13], as well as in diseases linked to metabolic syndrome, including atherosclerosis [14], ischemic stroke-induced inflammation [15], and chronic liver diseases [16]. Additionally, IL−1β upregulation is linked to iNOS induction via nuclear factor-kappa B (NF-κB) activation, which works synergistically with TNF-α to drive significant iNOS overexpression [17,18]. IL−1β upregulation is also linked to the release of nitric oxide (NO) by pancreatic β-cells, causing β-cell death [19]. NO, in turn, can activate IL−1β creating a positive feedback loop between IL-1β and iNOS—a “vicious cycle” that drives chronic inflammation and tissue damage [20,21].

The increase in the tissue expression of iNOS and the presence of infiltrating inflammatory cells (CD45-positive) are key biomarkers that allow researchers and clinicians to monitor inflammatory processes, as they offer insight into the progression and status of inflammation [22,23]. For example, (i) in inflammatory bowel diseases, pro-inflammatory cytokines are associated with the upregulation of iNOS [22]; (ii) pro-inflammatory cytokines are also associated with the upregulation of infiltrating inflammatory cells in type 2 diabetes mellitus, inducing chronic inflammation and insulin resistance [24]; and (iii) upregulation of iNOS and inflammatory cell infiltration are linked to liver fibrosis induction via different mechanisms [25,26]. Furthermore, in chronic liver injury, p53 accumulates in damaged hepatocytes, and the sustained activation and accumulation of p53 promote liver fibrosis, whereas in the initial stages of acute liver injury, p53 promotes an anti-fibrotic effect, which helps limit the fibrotic process [27].

Quercetin is a polyphenol with significant pleiotropic properties, acting as a potent anti-inflammatory and antioxidant compound. It is highly abundant in onions, apples, and berries [28]. Quercetin has been shown to combat chronic diseases, reduce hepatic steatosis and liver injury [29], and protect pancreatic β-cells while ameliorating acute pancreatitis [30,31]. These effects are largely attributed to scavenge free radicals and modulate inflammatory signaling pathways [32,33]. Previously, there was no single Federal Drug Administration (FDA)-approved drug specifically for NAFLD [34]. However, FDA recently approved the first drug to treat the complicated cases of NAFLD (NASH with liver fibrosis). Therefore, this study was designed to investigate a potential association between NAFLD/MASLD and the hepatic IL-1β/iNOS and IL-1β/CD45 axes of liver inflammation and fibrosis, as well as the status of dyslipidemia, hyperglycemia, and apoptosis with and without quercetin treatment.

## 2. Materials and Methods

### 2.1. Experimental Design

This work follows the ARRIVE guidelines, and all experimental rat procedures were endorsed by the ethical committee at Cairo University (CU/III/F/17/23; dated March 2023). Rats were kept in a clean facility at room temperature with a cycle of 12 h light/dark, and had free access to water and food. Following the acclimatization period, rats were assigned identification numbers and randomized using Excel software. All procedures were carried out in the animal house by experienced technicians under the supervision of a responsible veterinarian. Sample size estimation was conducted with G*power software, version 3.1.9.7. Data collection, analysis, and assessment were conducted in a blinded manner. In total, 24 rats (Wistar male, weight 170–200 g per animal) were equally allocated into 3 groups; (i) control group (Control) rats were fed with standard laboratory chow for 4 weeks and received during these period daily saline (vehicle) injections; (ii) model group (NAFLD) rats were fed with a HFCD diet [35] for 4 weeks and received during these period daily saline (vehicle) injections; and (iii) quercetin-treated (NAFLD-QUR) group rats were given daily injections of quercetin (50 mg/kg, i.p) [36] (Sigma Chemical Company, St Louis, MO, USA) and were fed the HFCD for 4 weeks. Then, blood was collected under anesthesia (sodium pentobarbital 50 mg/kg; EPICO, Tenth of Ramadan City, Egypt), before sacrificing the rats by cervical dislocation. Liver tissue samples were then harvested.

### 2.2. Determination of Blood Levels of Lipids, Glucose, Insulin, hs-CRP, Alanine Aminotransferase (ALT), and Aspartate Aminotransferase (AST)

Blood levels of free fatty acids (FFA), high-density lipoprotein (HDL), and hs-CRP were assessed using ELISA kits purchased from MYBioSource, Giza, Egypt, Cat No. MBS266907, MBS266554, and MBS8807983, respectively. Total cholesterol analysis was performed using kit # K4436-100 supplied by biovision, Cairo, Egypt. Serum glucose glucose was assessed using a kit purchased from Randox Laboratories Ltd., Crumlin, UK. Insulin level was assessed using ELISA kit retrieved from Invitrogen, thermofisher. Scientific, Waltham, MA, USA. Insulin resistance was determined using the HOMA-IR index. The formula is: as follows HOMA-IR = glucose (mmol/L) × fasting insulin (uIU/mL)/22.5 [37]. ALT and AST blood levels were measured using ELISA kits purchased from BioMed diagnostics (Badr city, Egypt). We followed the manufacturer’s instructions in all these measurements.

### 2.3. Determination of Liver Tissue Levels of Triglycerhides, Glutathione, Superoxide Dismutase (SOD), IL-6, and TNF-α

Hepatic tissue levels of triglycerhides were assessed using colorimetric assay kits (MBS9719080, MYBioSource, Giza, Egypt). ELISA kits were purchased from MYBioSource, Giza, Egypt and used to assess glutathione, #MBS265966, SOD, #MBS266897, and IL-6, #MBS2021530. Meanwhile, hepatic TNF-α levels were assessed using ELISA kits ab236712, purchased from Abcam, Cambridge, UK. We followed the manufacturer’s instructions in all these measurements.

### 2.4. Hepatic p53 and TIMP-1 qPCR

As previously reported [38], qPCR was performed for TIMP-1 (GenBank code: L29512.1) (sense, 5′-GGTTCCCTGGGCATCACTGAG-3′; antisense, 5′-CTCAGTGATGCCCAGGGAACC-3′), p53 (NCBI Reference Sequence: NM_001429996.1) (sense, 5′-GCCTGACTCAGACTGACAGCC-3′; antisense, 5′-GGCTGTCAGTCTGAGTCAGGC-3′), and β-actin, GenBank code KJ696744, Forward: 5′-AGCCATGTACGTAGCCATCCA-3′ Reverse: 5′-TCTCCGGAGTCCATCACAATG-3′.

### 2.5. Assessment of Liver Pathology

As previously described [39], liver paraffin blocks were sectioned at 5 μm and subsequently deparaffinized, rehydrated, and then proceeded for immunohistochemical staining after the antigen retrieval process. Tissue slides were incubated overnight with anti-IL-1β antibody (1:200), anti-iNOS antibody (1:500), anti-α-SMA (1:200), and anti-CD45 antibody (1:100) (Abcam, Cambridge, UK), and then incubated with HRP-conjugated goat anti-rabbit IgG secondary antibody (Abcam, Cambridge, UK) for 30 min. Then, sections were treated with 3,3′ diaminobenzidine for visualization of immunoreactivity and counterstained with Meyer’s hematoxylin. Isotype controls yielded no background staining, supporting the specificity of the primary antibody binding. Liver sections were also stained with Masson’s trichrome to evaluate fibrosis. The mean area % of these immunostaining and collagen deposition was assessed in 6 non-overlapping fields (×40) for each group after calibrating the software to automatically translate the pixels into actual micrometer units using Image J software 1.x (Cambridge, UK). Data were tabulated and the mean was statistically analyzed. Brunt fibrosis staging system that classifies liver fibrosis into four stages: Stage 0: no fibrosis, Stage 1: perisinusoidal fibrosis, Stage 2: portal/periportal fibrosis, Stage 3: bridging portal–portal or portal–central fibrosis, and Stage 4: cirrhosis with significant nodule formation [40,41] was also determined by two independent pathologists.

### 2.6. Statistical Analysis

Results are presented as mean ± SD. Statistical analyses were performed using SPSS software (version 20; SPSS, Inc., Chicago, IL, USA). To ensure data normality, we applied the Kolmogorov–Smirnov and Shapiro–Wilk tests; both confirmed that the data followed a normal distribution. A one-way ANOVA was conducted, and significant effects were examined using Tukey’s post hoc test. The nonparametric Kruskal–Wallis and Mann–Whitney tests were used to analyze the data for the fibrosis score and presented as median and interquartile range (median; 25–75%). To evaluate potential associations between the two parameters, we used Pearson correlation analysis, applying a significance threshold of *p* ≤ 0.05.

## 3. Results

### 3.1. Induction of NAFLD Biomarkers Is Inhibited by Quercetin

**Figure 1:**
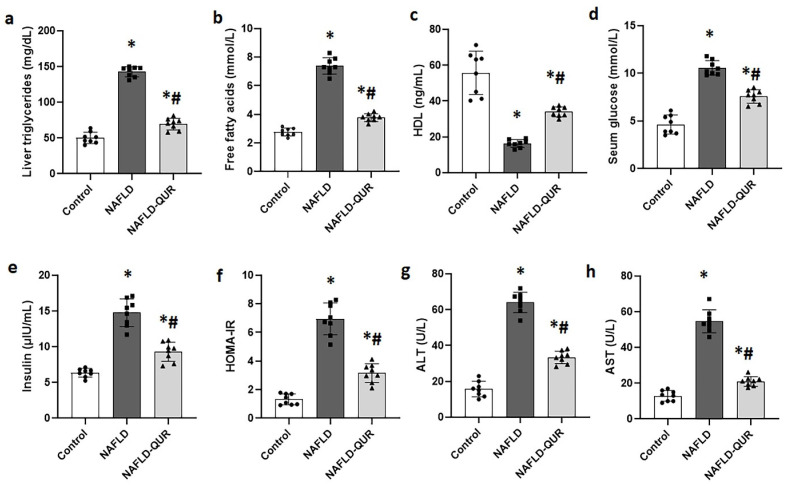
Quercetin protects against HFCD-modulated biomarkers of NAFLD and liver injury. Liver and blood levels of (**a**) liver triglycerides, (**b**) free fatty acids, (**c**) high-density lipoprotein, (**d**) glucose, (**e**) insulin, (**f**) HOMA-IR, (**g**) ALT, and (**h**) AST were assessed in all rats at the end of the experiment. Data are expressed as mean ± SD. *: significant compared to control group, #: significant compared to NAFLD group at *p* ≤ 0.004. HFCD: high-fat and high-carbohydrate diet; NAFLD: non-alcoholic fatty liver disease; HDL: high-density lipoprotein; HOMA-IR: homeostatic model for assessment of insulin resistance; ALT: alanine aminotransferase; AST: aspartate aminotransferase.

A diet high in both fat and carbohydrates is a recognized risk factor for developing NAFLD [42]. We modeled the disease in rats and assessed the association between blood and hepatic tissue levels of NAFLD biomarkers and the polyphenolic compound quercetin in all rats at day 29. As shown in the model group, HFCD caused a 2–5-fold increase in liver triglycerides (Figure 1a), free fatty acids (Figure 1b), glucose (Figure 1d), insulin (Figure 1e), HOMA-IR (Figure 1f), ALT (Figure 1g), and AST (Figure 1h). These changes were markedly reduced (*p* < 0.001) following treatment with quercetin (NAFLD-QUR). Quercetin treatment also augmented blood levels of HDL, which were reduced by NAFLD (Figure 1c). However, the protective effects of quercetin remained significantly elevated relative to the control group (*p* ≤ 0.004). This indicates that only partial protection by quercetin was achieved.

### 3.2. Quercetin Protects Against HFCD-Induced the IL-1β/iNOS Axis in Liver Tissue

**Figure 2:**
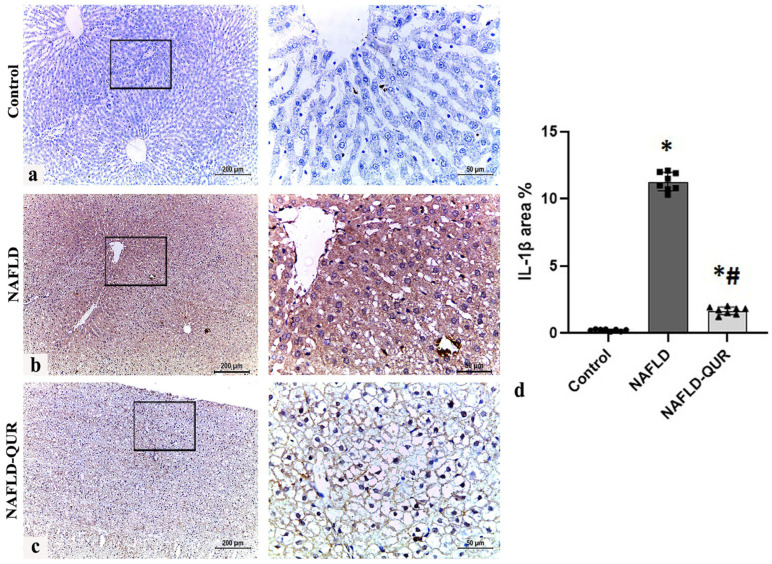
Induction of hepatic IL-1β by HFCD is inhibited by quercetin (QUR). Immunohistochemistry of IL-1β in liver sections obtained at the end of the experiment from the (**a**) Control, (**b**) NAFLD, and (**c**) NAFLD-QUR groups are depicted (scale bar 200 μm for low magnification (×100) and 50 μm for high magnification (×400)). (**d**) Area percentage of IL-1β immunohistochemical expression in liver sections from the above groups. Black squares represent selected areas that were examined at higher magnification (×400), as shown in the adjacent panels. Data are expressed as mean ± SD. *: significant compared to the control group, #: significant compared to the NAFLD group at *p* < 0.001. IL-1β: interleukin-1β; HFCD: high-fat and high-carbohydrate diet; NAFLD: non-alcoholic fatty liver disease.

**Figure 3:**
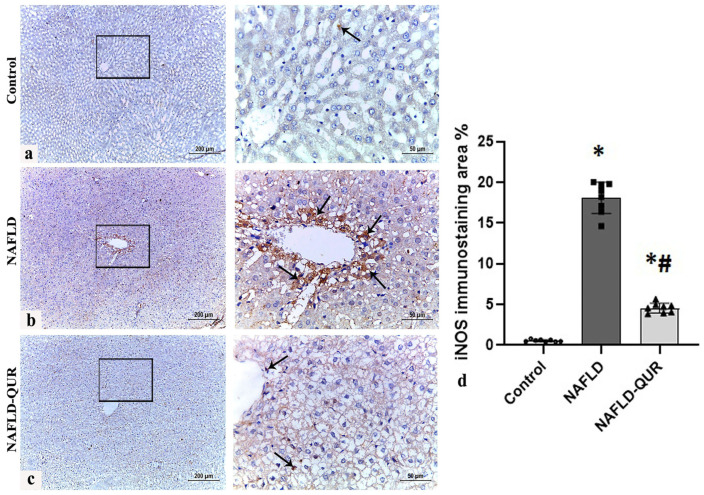
Induction of hepatic iNOS by HFCD is inhibited by quercetin (QUR). Immunohistochemistry of iNOS in liver sections obtained at the end of the experiment from the (**a**) Control, (**b**) NAFLD, and (**c**) NAFLD-QUR groups are depicted (scale bar 200 μm for low magnification (×100) and 50 μm for high magnification (×400)). (**d**) Area percentage of iNOS immunohistochemical expression in liver sections from the above groups. Black squares represent selected areas that were examined at higher magnification (×400), as shown in the adjacent panels. Data are expressed as mean ± SD. *: significant compared to the control group, #: significant compared to NAFLD group at *p* < 0.001. iNOS: inducible nitric oxide synthase; HFCD: high-fat and high-carbohydrate diet; NAFLD: non-alcoholic fatty liver disease.

Liver injury associated with the upregulation of IL-1β and iNOS, which exacerbate injury, in many liver diseases is well known [43]. Therefore, in view of the augmentation of liver injury enzymes in our model of NAFLD and the established connection between IL-1β and iNOS in metabolic dysfunction [44], we evaluated the hepatic IL-1β/iNOS axis in all groups with and without QUR (Figure 2 and Figure 3). Immunohistochemistry of NAFLD liver tissue performed at the end of the experiment showed a significant (*p* < 0.001) increase in IL-1β protein expression, with widespread cytoplasmic IL-1β immunohistochemical expression throughout the liver parenchyma (Figure 2b,d), compared to weak to negative expression in the control group (Figure 2a,d). Quercetin treatment (NAFLD-QUR) substantially decreased IL-1β expression (Figure 2c,d), although levels remained statistically superior to controls (*p* < 0.001).

We then evaluated iNOS protein levels induced by HFCD in livers harvested from all rats (Figure 3). Compared to negative to weak positive iNOS immunostaining in the control (Figure 3a,d), iNOS immunohistochemical staining of liver tissue sections from the model group (Figure 3b,d) revealed strong iNOS-positive cells in the periportal area (arrows). Quercetin treatment (NAFLD-QUR) substantially ameliorated iNOS expression (Figure 3c,d) although levels remained significantly elevated compared to controls (*p* < 0.001).

### 3.3. Quercetin Protects Against HFCD-Modulated CD45, Inflammation, Antioxidants, and p53 in Liver Tissue

**Figure 4:**
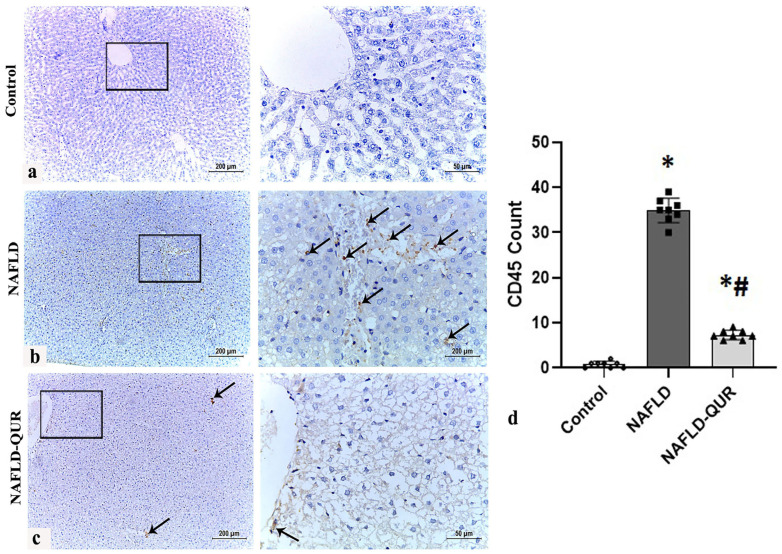
Induction of hepatic CD45 by HFCD is inhibited by quercetin (QUR). Immunohistochemistry of CD45 in liver sections obtained at the end of the experiment from the (**a**) Control, (**b**) NAFLD, and (**c**) NAFLD-QUR groups are depicted (scale bar 200 μm for low magnification (×100) and 50 μm for high magnification (×400)). (**d**) Area percentage of iNOS immunohistochemical expression in liver sections from the above groups. Black squares represent selected areas that were examined at higher magnification (×400), as shown in the adjacent panels. Data are expressed as mean ± SD. *: significant compared to the control group, #: significant compared to NAFLD group at *p* < 0.001. CD45: cluster of differentiation–45 (leukocyte common antigen); HFCD: high-fat and high-carbohydrate diet; NAFLD: non-alcoholic fatty liver disease.

IL-1β is located upstream of inflammatory cell infiltration (CD45+ve) [45], and there is a positive feedback loop between iNOS and inflammatory cell infiltration, creating a cycle that worsens tissue inflammation [46,47] leading to tissue damage and depletion of the body’s natural antioxidants [48]. Therefore, in light of our findings, we assessed hepatic CD45, inflammation, antioxidants, and apoptosis levels in all rats (Figure 4 and Table 1). HFCD caused a sharp increase in the number of CD45-positive cells (mean 34.9 ± 2.52) around the portal vein and hepatic sinusoids (arrows) in liver tissue sections from the model group (NAFLD) (Figure 4b,d), compared to an extremely low number of CD45-positive cells (mean 0.75 ± 0.71) in the control group (Figure 4a,d). Quercetin treatment (NAFLD-QUR) markedly but not completely (*p* < 0.001) decreased the number of CD45-positive cells (mean 7.25 ± 0.97) showing few interstitial and perivascular immunopositive cells (arrows) (Figure 4c,d).

The assessed hepatic antioxidants (glutathione and SOD), hepatic and blood inflammatory biomarkers (IL-6, TNF-α, and hs-CRP), as well as hepatic p53 are shown in Table 1. Compared to the control, the model group (NAFLD) demonstrated that HFCD triggered a significant reduction (*p* < 0.001) in antioxidant levels and an increase in inflammatory markers, which were partially protected by quercetin treatment (NAFLD-QUR). Quercetin was also associated with inhibition of the apoptotic biomarker p53, which was elevated by HFCD during the 28-day period.

### 3.4. Quercetin Protects Against HFCD-Induced Liver Fibrosis

**Figure 5:**
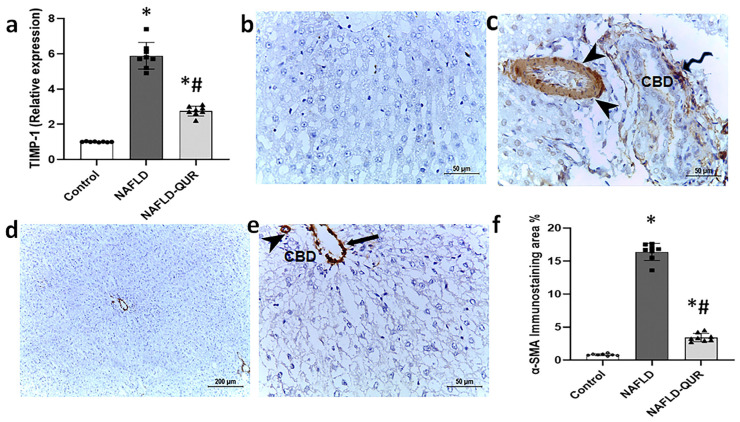
Induction of hepatic TIMP-1 and α-SMA by HFCD is inhibited by quercetin (QUR). (**a**) Relative expression of TIMP-1 mRNA in all animal groups. (**b**–**e**) Immunohistochemistry of α-SMA in liver sections obtained at the end of the experiment from the (**b**) Control, (**c**) NAFLD, and (**d**,**e**) NAFLD-QUR groups are depicted (scale bar 200 μm for low magnification (×100) and 50 μm for high magnification (×400)). (**f**) Area percentage of α-SMA immunohistochemical expression in liver sections from the above groups. Data are expressed as mean ± SD. *: significant compared to the control group, #: significant compared to NAFLD group at *p* < 0.001. TIMP-1: tissue inhibitor of metalloproteinases 1; α-SMA: alpha smooth muscle actin; HFCD: high-fat and high-carbohydrate diet; NAFLD: non-alcoholic fatty liver disease.

IL-1β downstream cell signalling pathways lead to fibrosis [49], and cardiac fibrosis is inhibited in p53 knockout mice [50]. In view of our results showing activation of the hepatic IL-1β axes and p53, we first assessed two biomarkers of fibrosis (TIMP-1 and α-SMA). TIMP-1 indirectly activates α-SMA secretion by mature myofibroblasts, as it promotes the conversion of hepatic stellate cells (HSCs) into myofibroblasts, which produce collagen and other matrix proteins that cause fibrosis [51,52]. Hepatic TIMP-1 gene expression markedly increased in the NAFLD group, and this was significantly (*p* < 0.001) but not completely inhibited by quercetin (NAFLD-QUR) treatment (Figure 5a). Immunohistochemistry of NAFLD liver tissue revealed significantly (*p* < 0.001) elevated α-SMA protein expression in activated myofibroblasts (spiral arrow) near dilated branches of the common bile duct (CBD), along with increased thickness of the vascular medial layer of the hepatic artery with myofibroblast deposition (arrowheads) (Figure 5c,f), compared to weak to negative α-SMA expression in the control group (Figure 5b,f). Quercetin treatment (NAFLD-QUR) substantially decreased α-SMA expression (Figure 5d–f). Despite this, levels maintained a significant elevation over controls (*p* < 0.001), as demonstrated by few positive α-SMA around the hepatic artery (arrowhead) and the portal vein (arrow) (Figure 5e).

**Figure 6:**
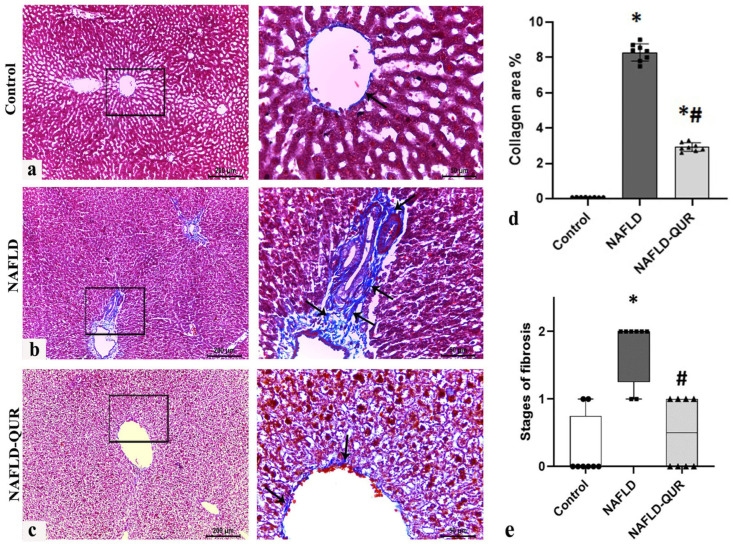
Induction of liver fibrosis by HFCD is inhibited by quercetin (QUR). Masson’s trichrome-stained images of liver sections obtained at the end of the experiment from the (**a**) Control, (**b**) NAFLD, and (**c**) NAFLD-QUR groups are depicted (scale bar 200 μm for low magnification (×100) and 50 μm for high magnification (×400)). Collagen (fibrosis) area % (**d**) and stages of fibrosis (**e**) in liver sections from the above animal groups. Black squares represent selected areas that were examined at higher magnification (×400), as shown in the adjacent panels. Data are expressed as mean ± SD. *: significant compared to the control group, #: significant compared to NAFLD group at *p* ≤ 0.002. HFCD: high-fat and high-carbohydrate diet; NAFLD: non-alcoholic fatty liver disease.

Masson’s trichrome-stained liver sections of the NAFLD group showed dense, thickened collagen bundles (arrows) around the portal vein, hepatic artery, and common bile duct (Figure 6b), compared to minimal collagen deposition in the control group (Figure 6a) (mean 8.29 ± 0.461 versus 0.085 ± 0.012; Figure 6d). Quercetin treatment (NAFLD-QUR) substantially (*p* < 0.001) decreased collagen deposition (mean 2.92 ± 0.22) with thin, regularly arranged fibrils (arrows) (Figure 6c,d). Additionally, mild to moderate fibrosis, as determined using the Brunt fibrosis staging system, was observed and significantly (*p* = 0.002) reduced by quercetin treatment to levels comparable to the control (*p* = 0.6) (Figure 6e).

### 3.5. Correlation Between IL-1β Score and Steatosis, Glycemia, iNOS, CD45, hs-CRP, ALT, α-SMA, and Fibrosis

**Figure 7:**
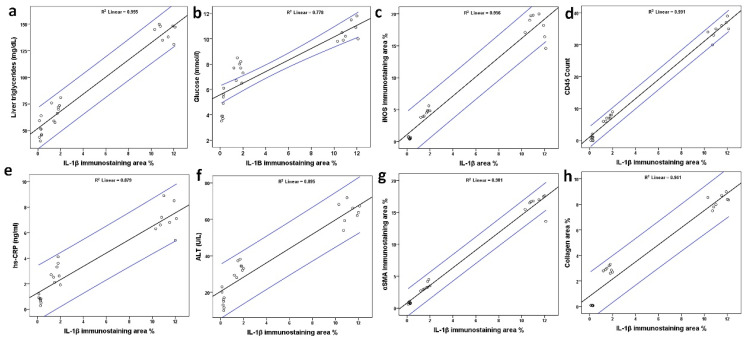
Pearson’s correlation data between the IL-1β and liver triglycerides (**a**), glucose (**b**), inducible nitric oxide synthase (iNOS) (**c**), inflammatory cell marker (CD45) (**d**), high-sensitivity C-reactive protein (hs-CRP) (**e**), alanine aminotransferase (ALT) (**f**), alpha smooth muscle actin (α-SMA) (**g**), and collagen deposition (**h**) are shown. The area between the two blue lines represents 95% confidence interval. Correlation is significant at the 0.01 level (2-tailed).

We further determined the correlation between IL-1β score and hepatic and blood levels of steatosis (liver triglycerides), glucose, iNOS, CD45, hs-CRP, ALT, α-SMA, and fibrosis (collagen deposition). This analysis demonstrates an association between the induction of the pro-inflammatory cytokine IL-1β and these parameters. IL-1β score showed a positive correlation with liver triglycerides (Figure 7a), glucose (Figure 7b), iNOS (Figure 7c), CD45 (Figure 7d), hs-CRP (Figure 7e), ALT (Figure 7f), α-SMA (Figure 7g), and collagen deposition (Figure 7h). A significant linear relationship between these variables was observed, with correlations significant at the 0.01 level (2-tailed).

## 4. Discussion

**Figure 8:**
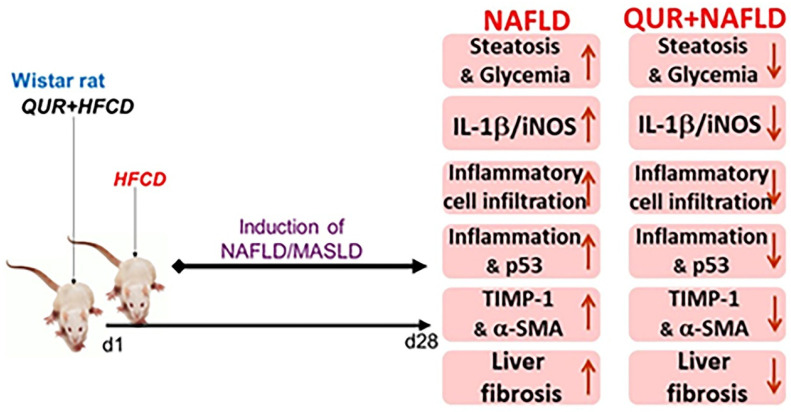
The proposed rat model for HFCD-induced NAFLD (left side) appears inhibited by quercetin (right side). ↑ = increased; ↓ = decreased. QUR: quercetin; HFCD: high-fat and high-carbohydrates diets; NAFLD: non-alcoholic fatty liver disease; MASLD: metabolic-associated steatotic liver disease; IL-1β: interleukin-1 beta; iNOS: inducible nitric oxide synthase; P53: tumor protein p53; TIMP-1: tissue inhibitor of metalloproteinases; α-SMA: alpha smooth muscle actin; d1: day one.

This study investigated the dysregulation of the hepatic IL-1β/iNOS and IL-1β/CD45 axes associated with the induction of liver inflammation, apoptosis, and fibrosis, as well as the depletion of hepatic intracellular antioxidants in an animal model of NAFLD/MASLD, with and without quercetin treatment (Figure 8). All these biochemical, molecular, and pathophysiological changes in the liver resulted from feeding rats a HFCD diet over 28 days, causing post-acute liver injury and liver fibrosis (stage 1–2), assessed on day 29, which may progress to more severe conditions over time if left unmanaged. Our data also demonstrated the beneficial, pleiotropic, protective effects of the flavonoid quercetin in this animal model. These findings also demonstrate an association between the cytokine IL-1β and these deleterious parameters (Figure 7).

The liver is a recognized target of obesity complications in humans, primarily through NAFLD/MASLD. Excess fat accumulation in the liver leads to inflammation, scarring, and potential damage, increasing the risk of severe liver disease, insulin resistance, and other cardiometabolic problems such as type 2 diabetes and heart disease [53,54]. Additionally, a systematic review and meta-analysis study demonstrated an association between NAFLD and the upregulation of the inflammatory biomarkers TNF-α, CRP, and IL-6 [55]. Moreover, downregulation of liver tissue levels of the antioxidants SOD and glutathione is reported in NAFLD patients, where the liver’s ability to fight oxidative stress is impaired [56], and depletion of the antioxidant glutathione induces apoptosis [57]. Chronic stress also induces the expression of the apoptotic marker p53 [58]. Our NAFLD/MASLD animal model data, which point to excess fat accumulation in the liver and hyperglycemia (Figure 1), induction of inflammation (hs-CRP, TNF-α, and IL-6) and apoptosis marker (p53), as well as inhibition of hepatic antioxidants (glutathione and SOD) (Table 1) and scarring (Figure 6), is in agreement with the above-mentioned reports.

Links are established between (i) IL-1β and NAFLD, with IL-1β acting as a key pro-inflammatory cytokine that promotes disease progression. Indeed, IL-1β contributes to NAFLD by disrupting both lipid and insulin signaling pathways, stimulating fatty liver (steatosis), and driving liver inflammation [59]. The transformation of NAFLD to NASH and liver fibrosis is reduced in IL-1β knockout mice [60]; (ii) iNOS and NAFLD, where studies show that iNOS expression is increased in NAFLD and appears to promote disease progression, partly by regulating macrophage function and inflammatory biomarkers (IL-1β, TNF-α, and IL-6) [61]. Additionally, biomarkers of liver injury (ALT and AST) were reduced in iNOS knockout mice in a mouse model of ischemia/reperfusion injury [62]; (iii) the activation of resident liver macrophages and the infiltration of inflammatory leukocytes, driven by lipotoxicity, lead to chronic inflammation, liver cell damage, and fibrosis [8]; and (iv) liver fibrosis and IL-1β [63], iNOS [25], and CD45, as it is a pan-leukocyte marker for immune cells involved in the inflammatory response that drives fibrosis [64]. These reports corroborate our own findings of an association between NAFLD and the induction of hepatic IL-1β, iNOS, CD45, inflammatory biomarkers, and liver fibrosis.

Furthermore, our data that point to the inhibition of hepatic IL-1β, iNOS, CD45, inflammation, apoptosis, liver injury enzymes, and liver fibrosis by quercetin are in line with previous studies on the (i) inhibition of IL-1β, IL-6, iNOS, ALT, and AST, as well as augmenting the antioxidants SOD and glutathione by quercetin in a mouse model of alcohol-induced liver injury [65]; (ii) inhibition of CD45 in paracetamol overdose-induced acute liver injury by quercetin and resveratrol [66]; (iii) inhibition of liver inflammation and fibrosis in a mouse model of carbon tetrachloride-induced chronic liver injury by quercetin [67]; and (iv) inhibition of oxidative stress and inflammation in a mouse model of NAFLD by quercetin [68].

## 5. Conclusions

In summary, we demonstrated the activation of hepatic IL-1β/iNOS and IL-1β/CD45 axes of inflammation and fibrosis, as well as the upregulation of p53 and biomarkers of NAFLD (dyslipidemia, hyperglycemia, insulin resistance, and liver injury enzymes) and the downregulation of hepatic intracellular antioxidants in a rat model of NAFLD/MASLD. These deleterious effects were mitigated by quercetin treatment, suggesting a potential therapeutic benefit in humans. However, (i) further preclinical studies are required to evaluate the compound’s safety, toxicity, and efficacy before clinical application; and (ii) this model reflects early-stage steatohepatitis and mild fibrosis (stage 1–2), which may not fully recapitulate the complexity of advanced human NASH or cirrhosis and requires further studies using long-term models.

### Limitations of the Study

The absence of a second control group treated with quercetin represents a limitation, as it prevents the study from demonstrating the baseline metabolic effects of quercetin. Furthermore, the route of administration (intraperitoneal injection rather than oral delivery) is another limitation, although gastrointestinal absorption of quercetin is generally low, with bioavailability estimated to be less than 17% in rats [69].

Additionally, the presence of condensed nuclei observed in the quercetin-treated group in the current study warrants further investigation, particularly in light of the role of quercetin in modulating apoptosis and cellular stress. Future in-depth histomorphological studies are recommended to further elucidate the structural remodeling induced by quercetin in liver tissue beyond steatosis improvement.

Finally, potential confounding variables cannot be excluded in the correlation analysis between IL-1β scores and the pathophysiological parameters of NAFLD, as specific molecular or pharmacological inhibitors were not utilized.

## Figures and Tables

**Table 1:** Effects of quercetin (QUR) on NAFLD-modulated antioxidants, inflammation, and apoptosis markers. Hepatic levels of glutathione, SOD, TNF-α, IL-6, and p53, as well as blood levels of hs-CRP were assessed at the end of the experiment in all rats. Data are expressed as mean ± SD. *: significant compared to the control group, #: significant compared to the NAFLD group at *p* ≤ 0.002. NAFLD: non-alcoholic fatty liver disease; SOD: superoxide dismutase; hs-CRP: high-sensitivity C-reactive protein; TNF-α: tumor necrosis factor alpha; IL-6: interleukin 6 and P53: tumor suppressor protein expression.

	Control	NAFLD	NAFLD-QUR
Glutathione (µg/mL)	83.38 ± 11	24.15 ± 6.28 *	63.73 ± 10.88 *,#
SOD (U/mL)	26.4 ± 3.47	7.83 ± 1.56 *	16.75 ± 1.49 *,#
hs-CRP (ng/mL)	0.75 ± 0.28	7.1 ± 1.07 *	2.85 ± 0.71 *,#
TNF-α (pg/mL)	14.64 ± 1.24	68.89 ± 6.64 *	35.61 ± 4.24 *,#
IL-6 (pg/mL)	8.03 ± 1.04	51.61 ± 6.52 *	22.55 ± 2.64 *,#
P53 (relative expression)	0.99 ± 0.01	8.27 ± 1.19 *	4.57 ± 0.43 *,#

## Data Availability

The data that support the findings of this study are available on request from the corresponding author.
